# Mapping cisplatin-induced viscosity alterations in cancer cells using molecular rotor and fluorescence lifetime imaging microscopy

**DOI:** 10.1117/1.JBO.25.12.126004

**Published:** 2020-12-16

**Authors:** Liubov E. Shimolina, Alexander A. Gulin, Miguel Paez-Perez, Ismael López-Duarte, Irina N. Druzhkova, Maria M. Lukina, Margarita V. Gubina, Nicolas J. Brooks, Elena V. Zagaynova, Marina K. Kuimova, Marina V. Shirmanova

**Affiliations:** a Privolzhsky Research Medical University, Institute of Experimental Oncology and Biomedical Technologies, Nizhny Novgorod, Russia; b Lobachevsky State University of Nizhny Novgorod, Nizhny Novgorod, Russia; c N.N. Semenov Federal Research Center for Chemical Physics Russian Academy of Sciences, Moscow, Russia; d Lomonosov Moscow State University, Department of Chemistry, Moscow, Russia; e Imperial College London, Faculty of Natural Sciences, Department of Chemistry, London, United Kingdom; f Moscow Institute of Physics and Technology, Dolgoprudny, Russia

**Keywords:** microviscosity of plasma membrane, fluorescent molecular rotors, fluorescence lifetime imaging microscopy, cancer, cisplatin, drug resistance

## Abstract

**Significance:** Despite the importance of the cell membrane in regulation of drug activity, the influence of drug treatments on its physical properties is still poorly understood. The combination of fluorescence lifetime imaging microscopy (FLIM) with specific viscosity-sensitive fluorescent molecular rotors allows the quantification of membrane viscosity with high spatiotemporal resolution, down to the individual cell organelles.

**Aim:** The aim of our work was to analyze microviscosity of the plasma membrane of living cancer cells during chemotherapy with cisplatin using FLIM and correlate the observed changes with lipid composition and cell’s response to treatment.

**Approach:** FLIM together with viscosity-sensitive boron dipyrromethene-based fluorescent molecular rotor was used to map the fluidity of the cell’s membrane. Chemical analysis of membrane lipid composition was performed with time-of-flight secondary ion mass spectrometry (ToF-SIMS).

**Results:** We detected a significant steady increase in membrane viscosity in viable cancer cells, both in cell monolayers and tumor spheroids, upon prolonged treatment with cisplatin, as well as in cisplatin-adapted cell line. ToF-SIMS revealed correlative changes in lipid profile of cisplatin-treated cells.

**Conclusions:** These results suggest an involvement of membrane viscosity in the cell adaptation to the drug and in the acquisition of drug resistance.

## 1 Introduction

Viscosity, a reciprocal of fluidity, plays an important role in the functioning of living cells, as it is one of the key parameters for the regulation of their morphological and physiological state. In particular, the viscosity of the cytoplasmic membrane is thought to be involved in controlling the transport of molecules, permeability, biosynthesis, and catalytic activity of membrane enzymes.^1^

Membrane viscosity depends on both lipid composition and organization. For example, a lower degree of fatty acid unsaturation, greater length of phospholipid acyl chains, and increased cholesterol content make a more viscous membrane.^1^^,^^2^ In addition, the plasma membrane is highly heterogeneous, as suggested in the “lipid raft hypothesis,” whereby cholesterol-rich, highly ordered lipid “islands” act as organizing hubs for membrane-embedded proteins.^3^ The complex interplay between all these factors allow living cells to maintain membrane viscosity within narrow limits, specific to each cell type, which is key to cell homeostasis and survival.^3^^–^^7^

Chemotherapy remains one of the main types of treatment for cancer. However, the resistance of tumor cells to chemotherapeutic drugs presents a serious and unsolved problem limiting the efficacy of the therapy.^8^^,^^9^ Growing number of studies suggest that response of cancer cells to the drug largely depends on biophysical properties of plasma membrane.^10^^,^^11^ The link between membrane viscosity and cell response to chemotherapy includes a few aspects. First of all, a membrane is a physical barrier for therapeutic agents to enter the cell. Higher viscosity reduces diffusion of the drug through a membrane and is considered as one of the mechanisms of chemoresistance.^12^^,^^13^ Second, membrane viscosity has profound consequences on the activity of drug efflux pumps, like P-glycoprotein that expel many therapeutic compounds from the cell and contribute to multidrug resistance.^14^ Finally, an increase of the plasma membrane fluidity favors induction of the Fas death-receptor mediated apoptosis.^15^ At the same time, chemotherapeutic drugs themselves can change the plasma membrane’s viscosity, even if the membrane is not the primary target for the drug action, e.g., via direct the interaction with lipid bilayer or indirectly, e.g., via lipid peroxidation.^10^^,^^16^^,^^17^ However, the interconnections between membrane viscosity and cellular drug response are poorly characterized so far.

Cisplatin and other platinum-based chemotherapeutics are the most widely prescribed drugs in modern oncology, administered to about 50% of all cancer patients.^18^^,^^19^ Their anticancer activity is primarily associated with the formation of platinum-DNA adducts that interfere with DNA replication and transcription and eventually result in suppression of cell proliferation and in induction of cell death. Meanwhile, interaction with DNA does not fully explain all cellular effects of cisplatin. It is now recognized that plasma membrane composition and fluidity affect cell sensitivity to cisplatin,^16^^,^^20^ whereas the effects of cisplatin administration on biological membranes have been explored very poorly. Although considerable research has been devoted to the interaction of cisplatin with lipid bilayers on the level of model membranes,^21^ rather less attention has been paid to cisplatin-induced changes in biophysical properties of membranes of cancer cells. Lacour et al.^22^ and Rebillard et al.^23^ reported on the rapid transient increase in plasma membrane fluidity in colorectal cancer cells exposed to cisplatin treatment, which was associated with initiation of apoptosis. To the best of our knowledge, these are the only two studies that address the relationship between cisplatin-induced changes in membrane viscosity in response to the drug.

Fluorescence lifetime imaging microscopy (FLIM) of fluorescent molecular rotors is a well established technique for measuring viscosity at the microscopic level (microviscosity).^24^^,^^25^ Molecular rotors are small synthetic viscosity-sensitive fluorophores, in which fluorescence lifetimes are strongly correlated to the microviscosity of their immediate environment.^24^^–^^27^ In a viscous medium, fluorescence lifetime increases, due to slowing down of the intramolecular twisting or rotation, which leads to a decrease in the nonradiative relaxation of the rotor’s excited state. Thus fluorescence lifetime of the rotor can be directly converted to the viscosity of its environment, using a previously recorded calibration curves in solvents of known viscosity (e.g., methanol–glycerol or toluene–castor oil mixtures).^25^ Notably, fluorescent molecular rotors allow both the spatially resolved quantitative imaging of viscosity, down to the resolution of individual cell organelles, and the dynamic measurements of viscosity in a living cell with high temporal resolution. Among molecular rotors, compounds based on boron dipyrromethene (BODIPY) have excellent sensitivity to the viscosity and are well suited for biological applications.^4^^,^^28^^–^^31^ Previously, we developed methodologies of the basis of FLIM to map the microviscosity of plasma membranes in individual cancer cells in a cell monolayer, 3D tumor spheroids, and mouse tumor *in vivo.*^4^^,^^29^^–^^31^

Time-of-flight secondary ion mass spectrometry (ToF-SIMS) is a surface sensitive technique that allows the detection and 3D visualization of organic compounds in cells with submicron spatial resolution.^32^^–^^34^ ToF-SIMS is mainly oriented on the analysis of lipids and small metabolites due to the high energy of primary ions. High chemical specificity and sensitivity make ToF-SIMS a valuable tool to detect unlabeled lipid species within plasma membrane. The method has demonstrated applicability for lipidomic analysis for various cancer cells.^35^^–^^37^

The aim of this work was to study cisplatin-induced changes in plasma membrane viscosity using FLIM with BODIPY-based molecular rotor in cancer cells in cell monolayer and multicellular spheroids and correlate them lipid profile, measured with ToF-SIMS, and responsiveness of cells to the treatment. The specific questions we addressed here are: (1) does cisplatin cause any changes in viscosity of cellular plasma membranes when used at therapeutically relevant concentrations; (2) how common are these effects for different cancer cell lines cultured as a monolayer and in multicellular structures; (3) are these changes associated with cell survival and/or cell death; and (4) if membrane lipid composition is the underlying reason for the observed changes in viscosity.

## 2 Materials and Methods

### 2.1 Cell Cultures and Treatment

HeLa Kyoto (human cervical cancer) and CT26 (mouse colorectal cancer) cells were used in the study. The cells were cultured in DMEM containing 100  μg/mL penicillin, 100  μg/mL streptomycin sulfate, and 10% fetal bovine serum at 37°C in a humidified atmosphere with 5% ${\mathrm{CO}}_{2}$.

To generate tumor spheroids, the HeLa Kyoto cells were seeded into ultra-low attachment 96-well round bottom plates (Corning Inc.), ∼100  cells/well in 200  μL DMEM (Life Technologies) and cultured in standard conditions (37°C, 5% ${\mathrm{CO}}_{2}$, and 80% humidity). In three days, spheroid formation was verified with light microscopy.

Chemotherapy was performed with cisplatin (Teva, Israel) at dose 2.57  μM (IC50) for CT26 cells and 1.1  μM (1/2 IC50), 2.3  μM (IC50), and 4.5  μM (2 IC50) for HeLa Kyoto cells grown as monolayer. The IC50 concentrations were determined in our previous experiments using MTT assay.^38^^,^^39^ HeLa spheroids were treated with 8.6  μM cisplatin. The tested concentrations of cisplatin were in the range of maximum concentrations of the drug in the blood plasma of patients, 1 to 2  mg/L or 3 to 6  μM on average.^40^ The cells were incubated with the drug from 10 min to 48 h, spheroids—from 3 to 48 h, and viscosity was measured immediately after the treatment. Untreated cells or spheroids served as a control. Additional experiment was performed to measure viscosity in monolayer cells in 48 h after therapy withdrawal.

The method for establishing the cisplatin-resistant cell subline was adopted from Ref. 41. Briefly, a HeLa cell culture was continuously exposed to gradually increasing concentrations of cisplatin. The start concentration was 1/150 IC50. Each next concentration was increased by 25% from the previous one and added only after clear adaptation of the cells to the drug, i.e., after restarting of cell proliferation without significant cell death in a plate (after 2 to 7 days). In ∼4 months after first exposure, the cells were considered cisplatin-resistant. Measurements of microviscosity in these cells were performed in 48 h after washing out the drug to avoid immediate effects of cisplatin.

### 2.2 Cellular Viability Assay

A live/dead double staining kit (Sigma)—calcein and propidium iodide (PI)—was used to stain live and dead HeLa Kyoto cells, respectively, after chemotherapy of cells in monolayer or 3D spheroids, according to the manufacture’s protocol. Fluorescence of calcein was excited by an argon laser at a wavelength of 488 nm and registered in the ranges 500 to 570 nm. PI fluorescence was excited at a wavelength of 543 nm and registered in the range 600 to 700 nm. One-photon fluorescence confocal images were obtained using an LSM 880 (Carl Zeiss, Germany) inverted laser scanning microscope.

### 2.3 Fluorescent Molecular Rotor BODIPY 2

BODIPY 2, 4,4-difluoro-4-bora-3a,4a-diaza-s-indacene (Fig. S1 in Supplementary Material), was synthesized according to the previously published procedure^28^ and was used as a viscosity-sensitive probe. This molecular rotor has already demonstrated the sensitivity of its spectral characteristics to viscosity, with a good dynamic range of fluorescence lifetimes.^28^ A calibration curve linking fluorescence lifetime of BODIPY 2 to the viscosity of its environment was previously obtained.^25^^,^^28^

An attractive property of the BODIPY 2 rotor is the selective staining of the plasma membrane, avoiding effective endocytosis. The effectiveness of measuring membrane viscosity was shown not only in cell cultures *in cellulo*,^4^^,^^28^^–^^31^ but also *in vivo* on animals with tumors.^30^ BODIPY rotors also have an excellent dynamic range of fluorescence lifetimes for the determination of viscosity in biologically relevant range, 1 to 5000 cP, which has been demonstrated to be temperature independent.^42^^,^^43^

For microscopic imaging, the cells were seeded on glass-bottom dishes for confocal microscopy (FluoroDishes, Life Technologies) overnight in complete DMEM media without phenol red (Life Technologies). The membrane viscosity was examined at 10 min, 1, 3, 6, 24, and 48 h after adding cisplatin. Before imaging, the culture media with cisplatin was replaced with ice-cold Hank’s solution without ${\mathrm{Ca}}^{2+}/{\mathrm{Mg}}^{2+}$, and cells were incubated at +4°C for 5 min. Afterward, Hank’s solution was replaced with ice-cold BODIPY solution (4.5  μM, 0.1% DMSO).

The spheroids were stained with BODIPY 2 on the day 5 of the growth, when they had a compact heterogeneous structure and the size of ∼350  μM. The five to six spheroids were carefully transferred on each glass-bottom dish (FluoroDishes, Life Technologies) in 1 mL DMEM without phenol red and placed in ${\mathrm{CO}}_{2}$ incubator for 2 h to allow their attachment. The membrane viscosity was examined at 3, 6, 24, and 48 h after adding cisplatin. Before imaging, the culture media with cisplatin was replaced with ice-cold Hank’s solution without ${\mathrm{Ca}}^{2+}/{\mathrm{Mg}}^{2+}$, and cells were incubated at +4°C for 10 min. Afterward, Hank’s solution was replaced with ice-cold BODIPY 2 solution (8.9  μM, 0.1% DMSO). Two-photon excited FLIM images were acquired within 5 to 10 min after adding BODIPY 2.

### 2.4 Large Unilamellar Vesicles

Lipids 1,2-dioleoyl-*sn*-glycero-3-phosphocholine (DOPC), cholesterol, and egg yolk sphingomyelin (EYSM) were purchased in powder form from Avanti Polar Lipids^®^ and resuspended in chloroform (20 mM) before use. A dried lipid film was created by mixing the lipid stock solutions at the appropriate DOPC:EYSM:cholesterol molar ratio before using a rotary evaporator to remove the solvent and to create a lipid film. 0.4 M aqueous sucrose solution was then added to hydrate the film to a final concentration of 1 mM lipid. The mixture was then vortexed and extruded 21 times through a 200-nm polycarbonate filter (Avanti Polar Lipids^®^). Lipid vesicles were then doped with an aliquot of BODIPY 2 (3 mM in DMSO), which was externally added to achieve 1:200 dye:lipid ratio. The final concentration of DMSO was 0.5% v/v. Solvents for fluorescence studies were of spectrophotometric grade (Sigma Aldrich^®^ or VWR). The mixtures were incubated for at least 15 min above the melting temperature of the lipid ($T_{m}$). After cooling, 200  μL large unilamellar vesicles (LUVs) were diluted in 1750  μL 9  mg/mL NaCl solution before measuring.

For cisplatin measurements, 50  μL of 180  μM cisplatin solution in 9  mg/mL NaCl was added to the LUV dilution described above and incubated in the dark at room temperature for 1 h before measuring.

All samples were placed in quartz cuvettes (10 mm path length). Time resolved fluorescence decay traces of BODIPY 2 were acquired using a Horiba Jobin Yvon IBH 5000 F time-correlated single-photon counting instrument with detection at 520±6  nm, after 404 nm pulsed excitation (NanoLED). Acquisition was stopped after peak counts reached 10,000 and the resulting traces were fitted to a biexponential decay using DAS^®^ software.

### 2.5 FLIM

Multiphoton tomograph MPTflex (JenLab, Germany) equipped with a tuneable 80 MHz, 200 fs Ti:Sapphire laser (MaiTai), a single-photon counting module SPC-150 and detector PMC-100-20 (Becker&Hickl, Germany) was used for FLIM. The images were acquired through a 40×, 1.3 NA oil immersion objective. The scan head of the MPTflex system was set up in the inverted position for *in vitro* imaging. The glass-bottom dish with cell monolayer of attached spheroids was placed on the adapter ring connected with a microscope objective. In the case of spheroids, the images were acquired from a depth of ∼20  μm.

BODIPY 2 fluorescence was excited at the wavelength of 800 nm and detected in the range 409 to 680 nm using a fixed prefitted emission filter. The average power applied to the sample was ∼7  mW. Image size was 512×512  pixels. The acquisition time was ∼7  s per image. The collected number of photons per the decay curve was at least 5000. FLIM images were obtained from 10 randomly selected fields of view in each culture dish.

Fluorescence lifetime analysis was performed in the SPCImage software (Becker&Hickl, Germany). Time resolved fluorescence decays at each pixel of the whole image were fitted using a monoexponential model. The goodness of the fit $\chi^{2}\leq 1.20$ indicated that the model used provided a reasonable fit. Fluorescence lifetime of BODIPY 2 (τ) was measured in the plasma membranes of cells by manual selecting zones with $\chi^{2}\leq 1.20$ as regions of interest.

The viscosity was calculated using the modified Förster–Hoffmann equation in the logarithmic form: $\log\;\tau_{f}=\alpha \;\log\;\eta +\text{const}$, where constant (α), log fluorescence lifetime ($\tau_{f}$) versus log viscosity (η). Experimentally measured lifetimes of BODIPY 2 (in ns) were converted to viscosity values (in cP) using the formula $x=y^{2}/0.0206$, where x—viscosity (in cP), y—fluorescence lifetime τ (in ns), on the basis of previously obtained calibration plot.^29^

### 2.6 ToF-SIMS

$5\times 10^{5}\text{ cells}$ were seeded on glass-bottom dishes (FluoroDishes, Life Technologies) containing clean and dry poly-L-lysine-coated cover glass and were incubated in an incubator at 37°C and an atmosphere of 5% ${\mathrm{CO}}_{2}$ for 24 h. The next day, cisplatin (2.6  μM) was added to the culture medium. After 20 min or 24 h incubation with cisplatin, cells were washed three times with phosphate-buffered saline (PBS) and then cells were incubated with 4% paraformaldehyde for 60 min at room temperature for chemical fixation. Afterward, cells were washed three times with PBS. In total, three samples were prepared—control sample without cisplatin and cells incubated with cisplatin for 1 and 24 h. Fixed cells were then stored in PBS for ∼5 to 6 h due to shipping to ToF-SIMS laboratory. Since ToF-SIMS analysis involves studies in vacuum, a cell dehydration procedure was applied. Cells were washed with mQ water to remove excess of salts. Drying was carried out under gentle stream of argon at room temperature.

Positive and negative mass-spectra were acquired with a ToF-SIMS 5 (ION-TOF Gmbh, Germany) equipped with a 30-keV ${{\mathrm{Bi}}_{3}}^{+}$ liquid metal ion source. Twelve mass spectra were recorded for each sample in both, positive and negative, ion mode, with an analysis area of $300\times 300\;{\mu m}^{2}$, and raster was 64×64  pixels. The primary ion dose density was $4\times 10^{11}\text{ ions}/{\mathrm{cm}}^{2}$ to maintain static SIMS conditions. A low-energy electron flood gun was used for charge compensation in all experiments. Ion yields were calculated as an intensity of the corresponding peak of interest normalized to the total ion count amount.

### 2.7 Statistics

The mean values (M) and standard deviations (SD) were calculated for the long and short components of fluorescence lifetimes of BODIPY 2. Student’s t-test was used to compare the data (p≤0.05 was considered statistically significant). The number of cells for mean value calculations was 20 to 30 in 7 to 10 fields of view.

## 3 Results

### 3.1 Viscosity in Monolayer Cells upon Cisplatin Treatment

Using FLIM with fluorescent molecular rotor BODIPY 2, plasma membrane viscosity in cancer cells was measured during chemotherapy with cisplatin that was applied to the cell monolayers in the clinically relevant concentrations. It is important to mention that membrane viscosity was measured only in viable cells, as in dead (PI positive) cells the rotor did not stain the plasma membranes, but accumulated inside the cells, where its fluorescence decayed biexponentially, indicative of rotor aggregation^44^ (Fig. S2 in the Supplemental Materials).

**Fig. 1:**
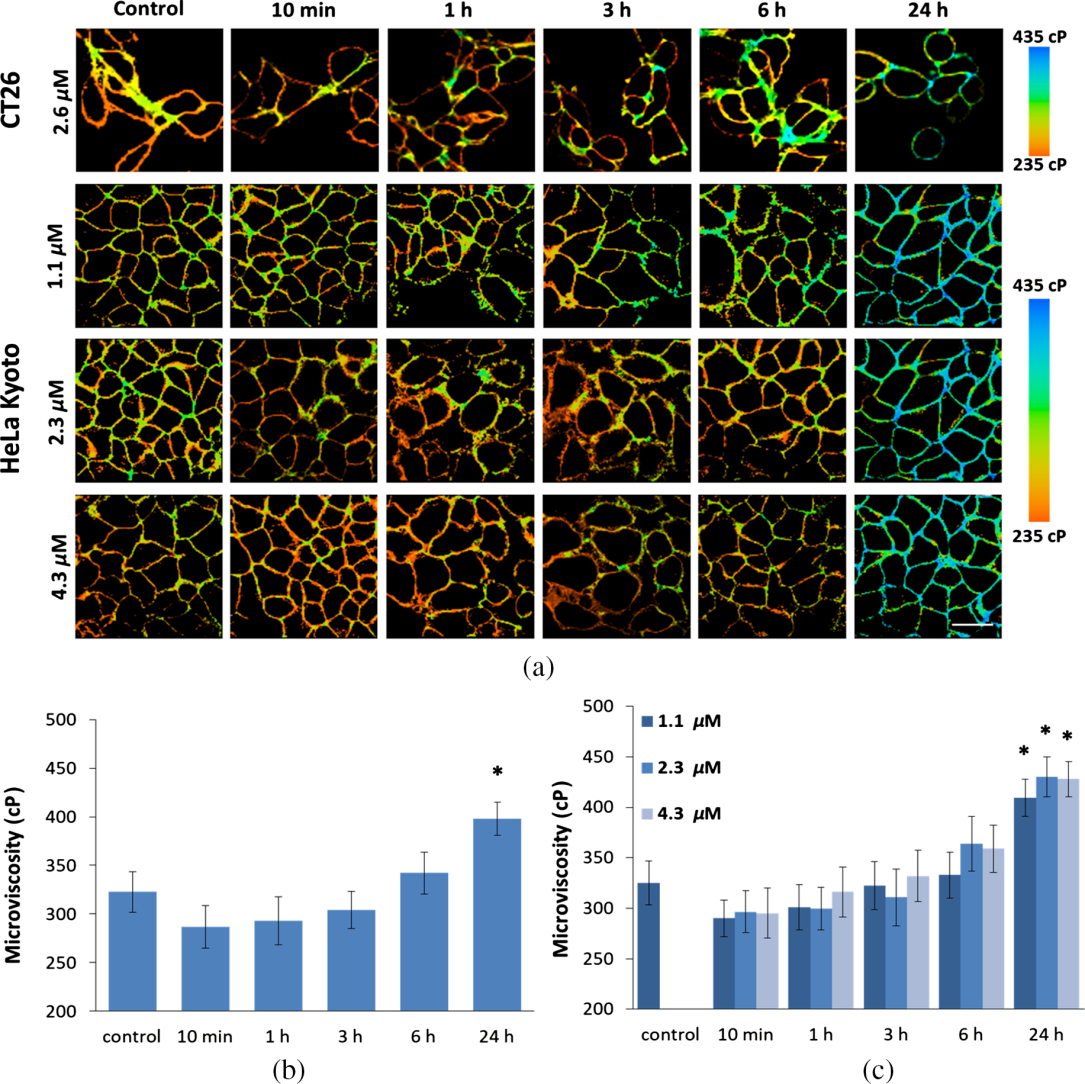
Plasma membrane microviscosity in monolayer CT26 and HeLa cells before (control) and during 24-h-exposure to cisplatin. (a) Viscosity images of cells acquired with FLIM. Quantification of viscosity of (b) CT26 and (c) HeLa cells during chemotherapy. Mean±SD, n=20 cells. Bar, 50  μm. *, p≤0.005 compare with control.

In untreated colorectal cancer CT26 cells, time-resolved fluorescence decays collected from plasma membrane locations could be fitted with monoexponential function, and fluorescence lifetime of BODIPY 2 was measured to be ∼2.55±0.07  ns, which corresponded to the viscosity value 322±21  cP. In the time period from 10 min to 3 h of incubation with 2.57  μM cisplatin, the membrane microviscosity decreased compared to the control and amounted to ∼290 to 300 cP (differences with control statistically not significant). At further incubation with cisplatin the viscosity increased and at 24 h time point became greater than the control value, 397±17  cP (p≤0.005) [Figs. 1(a) and 1(b) and Fig. S3 in the Supplemental Materials].

To find out whether cisplatin induces similar changes of viscosity in a different cell line and whether these effects depend on a drug dose, we have tested three doses of cisplatin (1.1, 2.3, and 4.5  μm) on cervical carcinoma HeLa cells [Fig. 1(a)]. Interestingly, no dose-dependent effects on viscosity were detected at the range of the doses we used, which likely indicates that the observed alterations in membrane viscosity are not associated with drug–lipids interactions that may occur as a result of a passive diffusion process but rather reflect the effect of a biophysical process that is identical in all treated cells at these treatment doses. Upon treatment of HeLa cells with cisplatin, a decrease in membrane viscosity was observed at early time points (10 to 60 min), when the viscosity was reduced from 326±22  cP to ∼300  cP (not significant). Starting from 6 h, the value of the membrane viscosity increased. At 24 h, the membrane viscosity was 427±26  cP [Fig. 1(c) and Fig. S3 in the Supplemental Materials].

We have further confirmed that cisplatin at 4.5  μM concentration (the highest concentration used in cells) does not have a direct effect on the lipid bilayer viscosity, by performing fluorescence lifetime measurements using BODIPY 2 in aqueous suspension of LUVs produced from DOPC, DOPC + cholesterol (50/50), and DOPC/Sphingomyelin/Cholesterol (25/25/50). The lifetime corresponding to the rotor embedded in the membrane increased in these series, from 1.8 to 2.6 ns and to 3.0 ns, as expected,^45^ due to an increased packing of lipids in the presence of cholesterol and sphingomyelin and the corresponding phase change from liquid disordered to liquid ordered lipid bilayer. However, the addition of cisplatin did not alter viscosity in any of these compositions (Fig. S4 in the Supplemental Materials). We note that the lifetime value (and viscosity) of DOPC/cholesterol (50/50) is very close to the lifetime value seen in colorectal cancer CT26 and in HeLa cells. The lack of viscosity change in LUVs upon incubation with a high concentration of cisplatin confirms that the effect of cisplatin on the plasma membrane viscosity is a consequence of its biological action on cellular function and not simply an effect of passive incorporation or association of this drug with the bilayer.

It is seen from PI- and DAPI-staining and bright-field microscopy (Fig. S2 in the Supplemental Materials) that most cisplatin-treated cells remained viable after 24 h, though their morphology was altered. After 24 h of incubation with cisplatin, the number of dead (PI-positive) HeLa cells increased to 7%, for CT26 cells the number of dead (PI- and DAPI-labeled) cells increased to 10%. As we previously showed, inhibition of cell proliferation, but not a cell death, is the main effect of cisplatin on HeLa cells upon the use of IC50 concentration over 24 h.^38^

To check how stable and persistent the observed viscosity changes are, we removed cisplatin from the culture medium after 24-h incubation and measured viscosity following additional 48 h of culturing. During this time, the cells did not recover their initial viscosity, but preserved the elevated viscosity of their plasma membranes (Fig. S5 in the Supplemental Materials).

### 3.2 Viscosity in Tumor Spheroids upon Cisplatin Treatment

Next, we assessed the plasma membrane viscosity of living cells within tumor spheroids during therapy with cisplatin. Spheroids are often used as an *in vitro* multicellular model of a solid tumor that mimics its basic structural and functional features and drug resistance profile. Previously, we have shown that microviscosity of plasma membranes in spheroid cells did not differ for spheroids of different sizes and between the inner (quiescent) and outer (proliferative) cellular layers of a spheroid.^29^ These data indicate that microviscosity of membrane is not affected by the metabolic nor proliferative activity of cells and by the heterogeneity of the cellular microenvironment in conventional conditions.

**Fig. 2:**
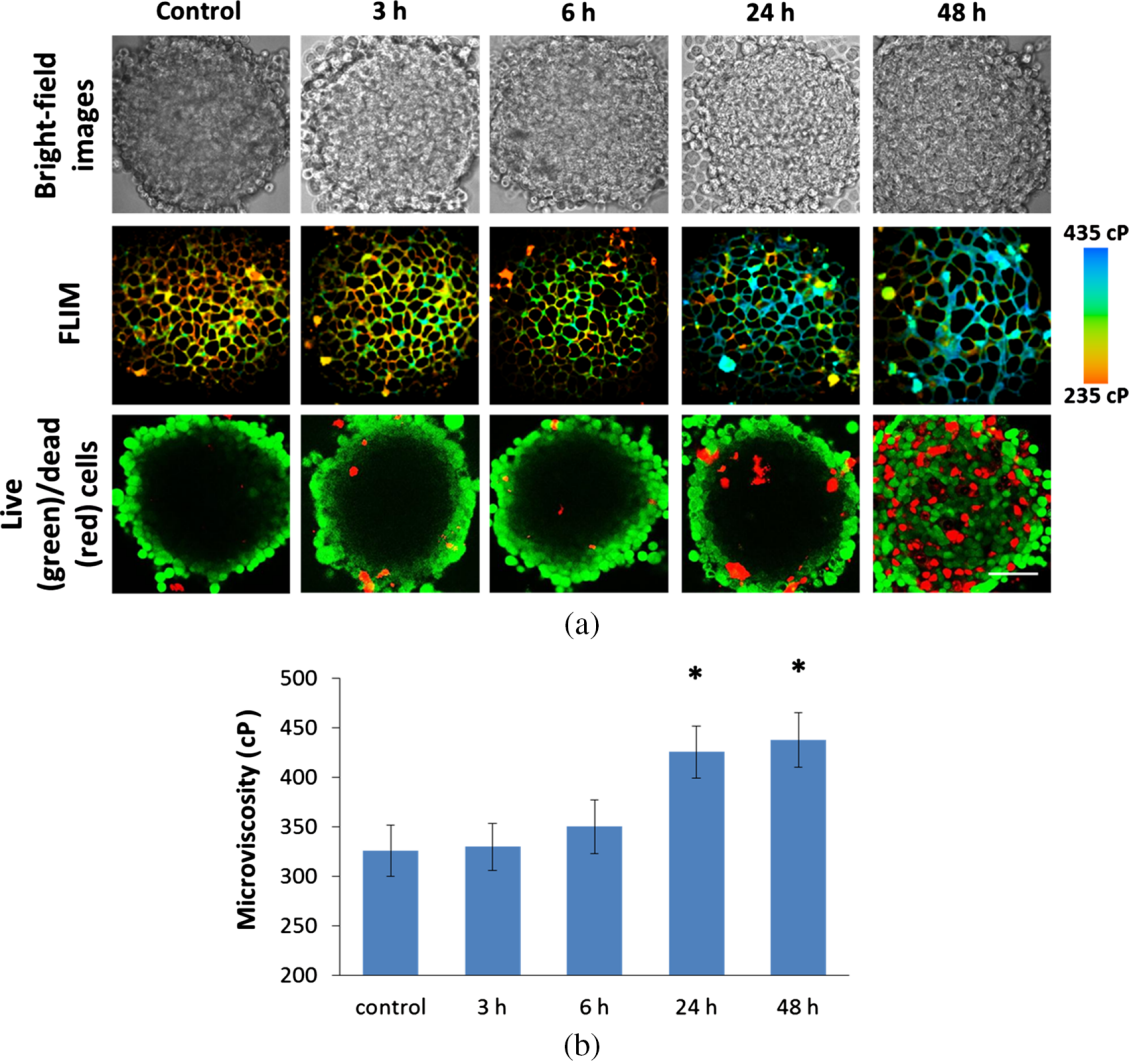
Plasma membrane microviscosity in tumor spheroids HeLa before (control) and during 48-h-exposure to cisplatin. (a) Bright-field microscopy, viscosity images of spheroids acquired with FLIM, and live (calcein)/dead (PI) cells assay. (b) Quantification of viscosity during chemotherapy. Mean±SD, n=4 spheroids, 20 cells in each. Bar, 80  μm. * Statistically significant difference with control, p≤0.005.

Membrane viscosity of HeLa cells in the tumor spheroids was identical to cells in a monolayer, 325±29  cP. Treatment with cisplatin resulted in a dramatic increase of the viscosity up to 425±25  cP after 24 to 48 h of incubation (p=0.0005) [Fig. 2(b)]. As expected, the treatment was accompanied by the increase in the number of dead cells, but the viscosity of viable cells did not change between 24 and 48 h. Moreover, we evaluated the changes in the morphology of spheroids upon incubation with cisplatin. For imaging, multicellular compact tumor spheroids of 5 days of growth, ∼350  μm in diameter, were taken, in which the outer layer of proliferating cells and the inner, more dense nucleus could be distinguished. After 48 h of incubation with cisplatin, the spheroid size increased to ∼450  μm (Fig. S6 in the Supplemental Materials). Although cisplatin treatment did not affect the size of the spheroids, an increase in the number of dead cells (PI-positive) and structural disorganization of the spheroids can be detected after 24- and 48-h exposure [Fig. 2(a)].

These experiments in cancer cell monolayers and in spheroids clearly showed that a pronounced increase in viscosity, observed at prolonged drug exposures (≥24  h) was not directly related to cell death because the viscosity changes observed were similar in all cells in a cell population, in those that died and in those that survived the treatment. It was also not a transient response to the treatment, because the increased viscosity persisted for at least 2 days after the drug withdrawal. This allows us to assume that increase in the membrane viscosity could be an early adaptive response to stress conditions (e.g., DNA damage), a part of cell defense mechanisms.

### 3.3 Viscosity in Cisplatin-Adapted Cells

**Fig. 3:**
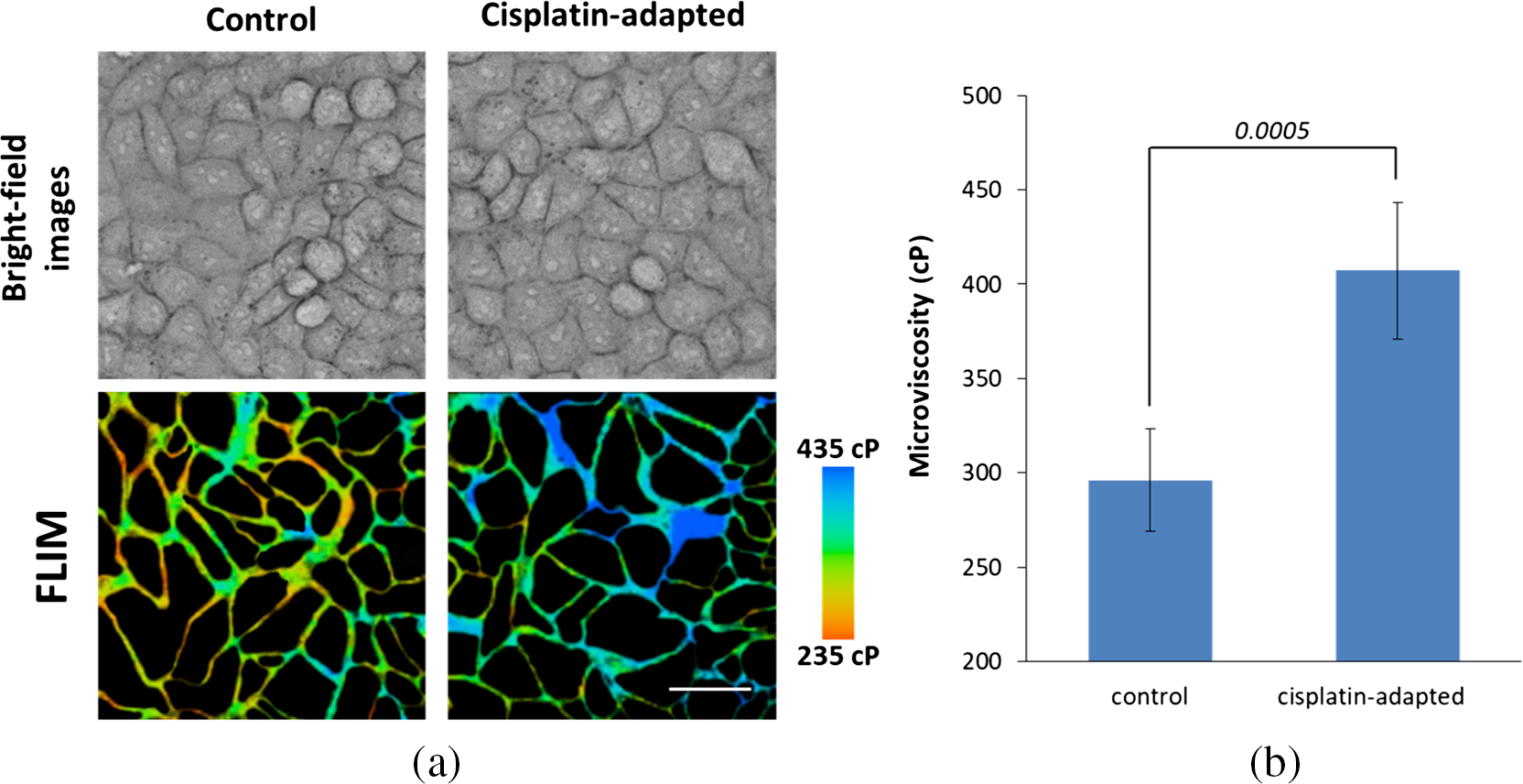
Plasma membrane microviscosity in cisplatin-adapted (resistant) HeLa cells. (a) Bright-field images and viscosity images of cells acquired with FLIM of fluorescent molecular rotor BODIPY 2. (b) Quantification of viscosity in control and resistant cells. Mean±SD, n=20  cells. Bar, 50  μm.

In line with the idea that increased membrane viscosity can be a consequence of physiological adaptation of cells to the drug, we analyzed viscosity in HeLa cell subline that had been adapted to cisplatin by prolonged (∼4 months) exposure to the drug. Drug-adapted cancer cell lines are commonly used as a preclinical model of acquired drug resistance.^46^^,^^47^ Cisplatin-adapted cells had the same morphology and proliferation rate as nonadapted counterpart, whereas the IC50 value increased from 2.3 to 6.26  μM, indicating that sensitivity to the drug decreased by 2.7 times. Using FLIM of BODIPY 2, we found that the viscosity of the plasma membrane in cisplatin-adapted cells was higher than in control cells, 407±38  cP versus 301±27  cP, p=0.0005 (Fig. 3 and Fig. S7 in the Supplemental Materials).

Noteworthy, the increase of membrane viscosity detected in both cisplatin-treated cells at late time-points and the cells adapted to the drug suggests that these changes in the membrane properties can favor the cell survival and contribute to the acquisition of drug resistance.

### 3.4 Lipid Composition of Plasma Membranes

**Fig. 4:**
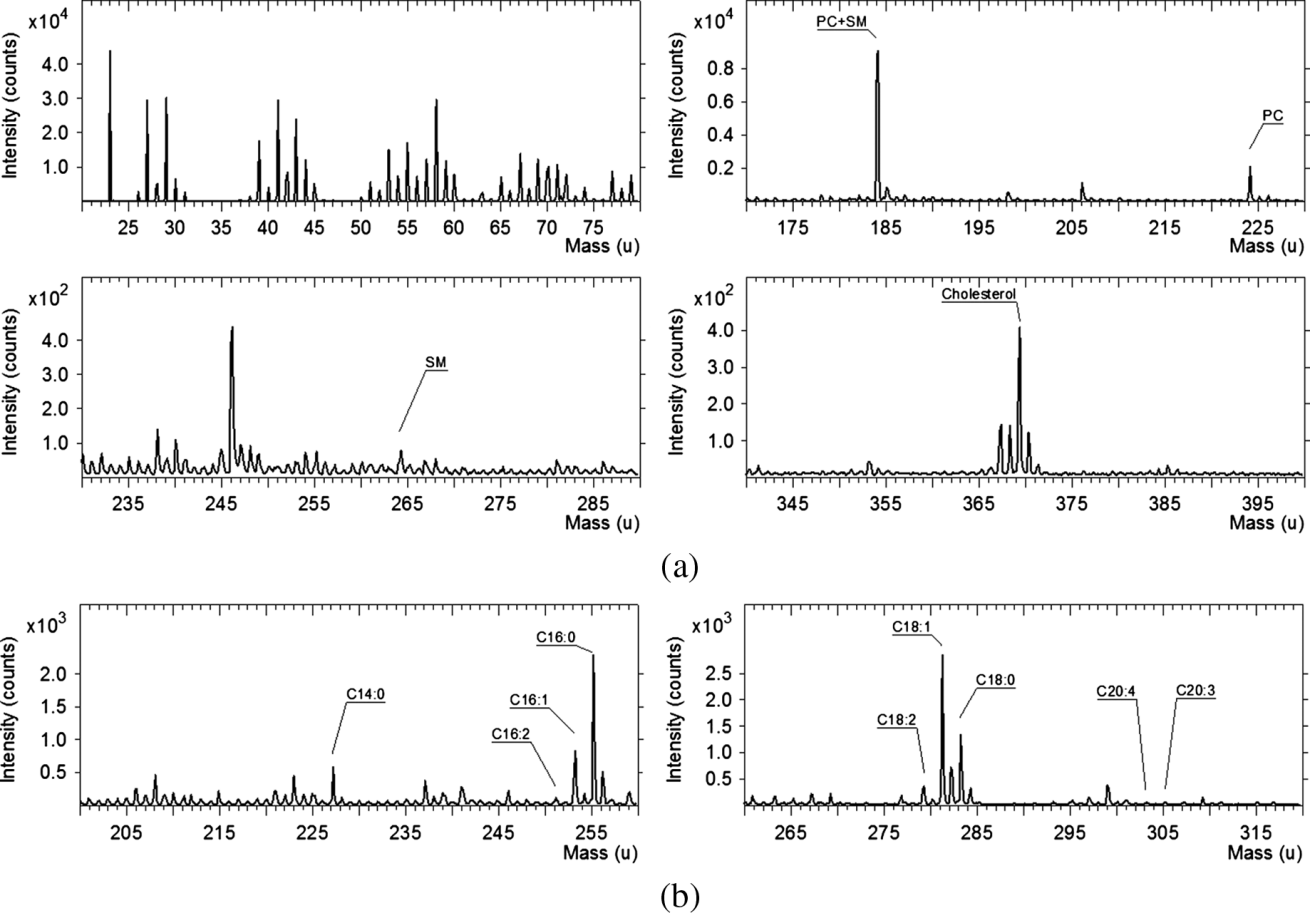
Representative mass spectra of CT26 plasma membranes: (a) positive ions and (b) negative ions. Fatty acids are designated as CX:Y, where X is the chain length and Y is a number of double bonds. PC, phosphatidylcholine and SM, sphingomyelin.

To determine whether the changes in plasma membrane viscosity during chemotherapy are caused by the alterations in the lipid composition, we obtained lipid profiles for CT26 cells upon cisplatin treatment using ToF-SIMS. Figure 4 shows typical mass spectra of CT26 cells plasma membranes. Labels show characteristic fragments and molecular ions. Since ToF-SIMS analyze only topmost layers of a sample (i.e., plasma membranes), fatty acids peaks [Fig. 4(b)] are likely originated as fragments of phospholipids. Phosphatidylcholine and sphingomyelin have similar chemical structure and therefore generate common peaks. Nevertheless, these species could be distinguished by m/z 224.1 and m/z 264.3 peaks that were utilized for analysis here. Lipid composition was analyzed at 1 and 24 h exposures to cisplatin, when the decrease and increase in viscosity were detected, respectively, and compared with untreated control.

**Fig. 5:**
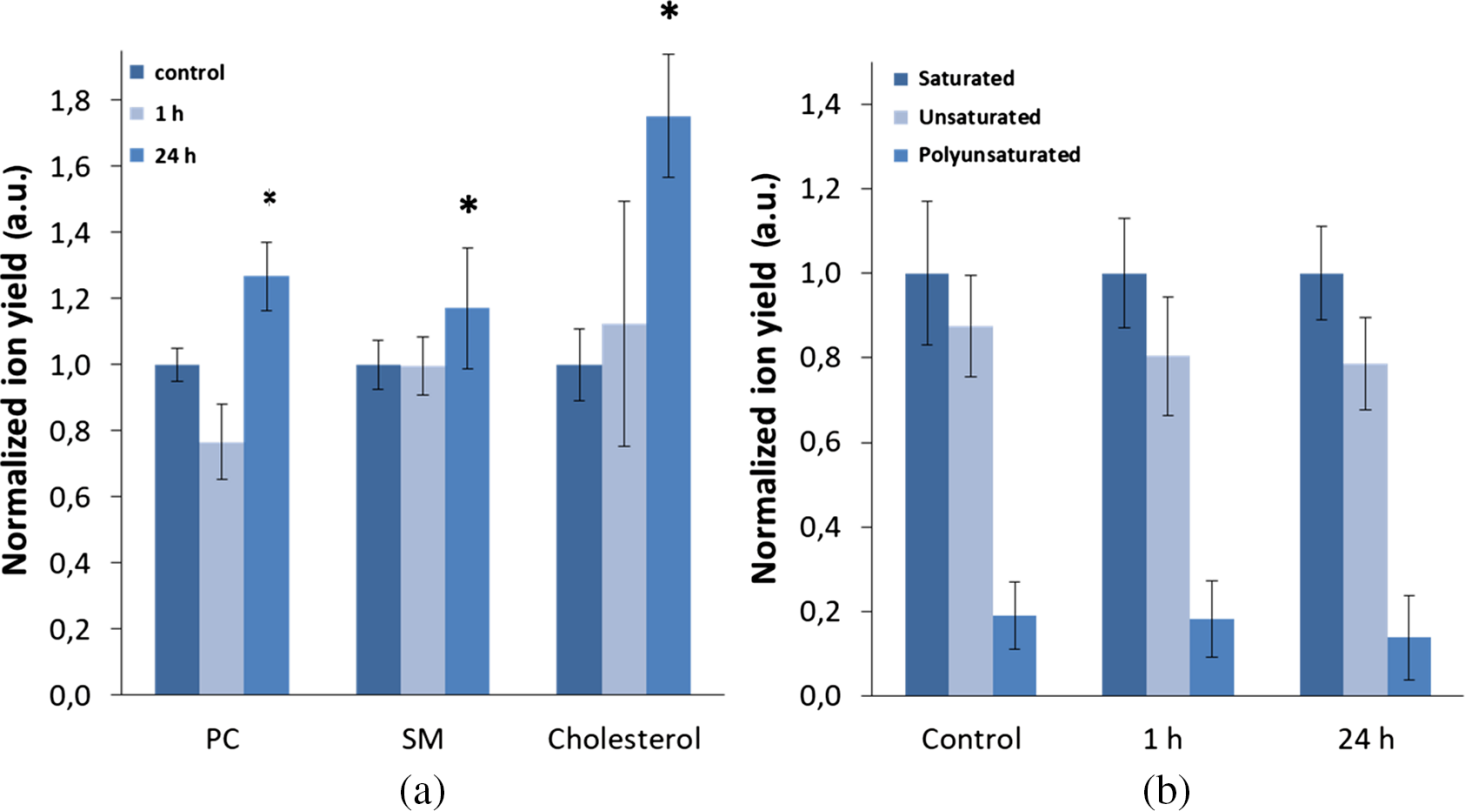
Lipid composition of CT26 plasma membranes obtained by ToF-SIMS: (a) positive ions. PC, phosphatidylcholine and SM, sphingomyelin. Data normalized to the control sample. (b) Fatty acids chains analysis in negative ions. Data normalized to saturated fatty acids. * Statistically significant difference with control, p≤0.001.

Figure 5 reveals ion yield variation of phosphatidylcholine (m/z 224.1), sphingomyelin (m/z 264.3), and cholesterol (m/z 369.3) ions for cisplatin exposed cells compared to a control sample. Sphingomyelin signal increases for 24 h administration compared with control and 1 h samples, which is in good accordance with detected increase in microviscosity. Interestingly, phosphatidylcholine intensity drops by more than 20% for 1 h of exposure and increases by more than 20% for 24 h of exposure to cisplatin. In addition, we recorded an increase in cholesterol peak intensity in the membranes of the treated cells during 24 h of incubation with cisplatin. Cholesterol is known as a regulator of membrane stiffness, therefore, the obtained data correlate well with the increase in viscosity that we registered using FLIM of BODIPY 2.

Figure 5(b) represents sum intensity of main peaks of saturated, unsaturated, and polyunsaturated fatty acids observed on mass spectra obtained in negative ion mode. For better presentation, data are normalized to signal of saturated fatty acids.

Unsaturated to saturated fatty acids ratio showed a tendency to decrease after cisplatin exposure. Although the effect is more pronounced after 24 h, it remained within statistical error. The ratio changes were mainly due to oleic acid signal variation. Polyunsaturated fatty acids also showed similar dynamic decrease, which is more pronounced for the 24-h sample.

## 4 Discussion

Here we used FLIM together with a fluorescent molecular rotor to investigate the effects of cisplatin on the viscosity of plasma membranes of cultured cancer cells. Our investigations have shown, for the first time that large increase in viscosity occurs at the conditions of prolonged treatment with and after adaptation to the drug, which may contribute to cell survival after therapy.

Despite the fundamental importance of viscosity for cell biology and physiology, its role in the pathogenesis of cancer and the response to therapy is not fully understood. Chemotherapy is one of the main treatments for malignant neoplasms. However, the physiological processes that occur when a chemotherapeutic agent acts on a living cell are not fully described. For example, cytoplasmic and membrane viscosity are modified when cancer cells are subjected to a chemotherapy process.^10^^,^^22^^,^^23^^,^^48^ In particular, effects of platinum-based drugs on cellular viscosity have been explored very poorly, which emphasizes the relevance of the study. Recent studies have shown that the response of a tumor to cisplatin is determined not only by the interaction of the drug with the primary target (nuclear DNA), but may also include multiple physiological and physicochemical changes.^23^

Previously, Lacour et al.^22^ and Rebillard et al.^23^^,^^49^ demonstrated that cisplatin treatment rapidly increased fluidity in bulk membranes as well as in lipid rafts in colorectal cancer cells HT29 between 15 min and 4 h after treatment. The authors showed that the early membrane fluidification occurs independently of and before cisplatin-DNA adduct formation and apoptosis triggered via Fas death receptor pathway. Their results suggest that the underlying mechanism of the increase in membrane fluidity is the activation of sphingomyelinase. Aggregation of Fas receptor crucially depends on the local lipid composition, specifically, on the sphingolipid ceramide. Some other apoptotic stimuli, including chemotherapeutic agents, induce generation of the sphingolipid ceramide that rapidly forms domains (rafts) within plasma membrane and facilitates clustering of the death receptors.^49^^,^^50^

In our study, we observed a small statistically insignificant decrease in membrane viscosity at early times following cisplatin treatment of monolayer cells, which was, however, reproducible and occurred in both cell lines studied—CT26 and HeLa Kyoto. According to some studies,^22^^,^^23^^,^^49^ fluidification of the plasma membrane is involved in induction of apoptosis. Our result on a minor fluidification is consistent with the absence of extensive apoptosis in these cell cultures at this time point. The essential difference between the above-mentioned and our studies is the drug concentrations used. We used a dose of cisplatin in the range from 1.1 to 4.5  μM for the monolayer cells, which is significantly less than in the above studies (5  μg/mL=16.6 and 25  μM, respectively).^22^^,^^23^^,^^49^ The choice of the drug dose in our study was based on the MTT-assay and a prior knowledge of concentration of cisplatin in the blood plasma of patients, which is 3 to 6  μM.^40^^,^^51^ Previously, we showed that cisplatin at a concentration of 2.4 to 2.6  μM affects the proliferative ability rather than viability of cancer cells HeLa—the number of dead cells did not exceed 10%, whereas the cells growth was inhibited after 24 h exposure to cisplatin.^38^

It is known that cisplatin increases the duration of S-phase and blocks cells in G2-phase of the cell cycle.^52^^,^^53^ It was previously suggested by fluorescence polarization studies that the viscosity of cellular membranes differs in different phases of the cell cycle: the maximum viscosity was achieved in mitosis, the minimum in the S phase.^54^ The cell cycle length for HeLa cells is ∼18 to 22 h^55^ and antiproliferative effects of cisplatin are evident at the same timescale. Based on these data, we conclude that the observed significant increase in viscosity in our experiments at 24 h cannot be explained by the cell cycle arrest alone.

The viscosity of cell membranes, in the absence of specific protein induced domains, is largely dependent on the qualitative and quantitative content of fatty acids in membrane lipids.^44^^,^^56^^,^^57^ Specifically, unsaturated fatty acids fluidify the membranes structure, whereas an increase in the cholesterol and saturated fatty acids content reduces membrane fluidity. Cholesterol plays an important role by increasing the packing of phospholipid molecules. In the liquid disordered phase, and it can also participate in the formation of lipid raft domains. Thus cholesterol is a stabilizer of plasma membrane viscosity. In addition to cholesterol, glycophospholipids that form the lipid bilayer determine the viscosity and permeability of membranes too. In our study, dramatic increase of cholesterol was established by ToF-SIMS after 24 h incubation of CT26 cells with cisplatin, which correlated well with significant increase in membrane viscosity detected by FLIM. Therefore, we suggest that cisplatin-induced changes in membrane viscosity are associated with the changes in lipid composition as revealed by mass spectrometry. Since ToF-SIMS was done with only one type of cells (CT26 line), we cannot exclude that in a different cell line the changes in the viscosity could be caused by a different change in membrane lipid composition.

Previously, cisplatin effects on membrane composition of A549 cells (human lung carcinoma) were studied by Zhang et al.^58^ A decreased phosphatidylcholine fraction and more abundant cholesterol signal were found in membranes of cisplatin-resistant cell, suggesting that the decreased membrane fluidity reduces cisplatin uptake.^58^ Gulin et al. showed cisplatin lateral distribution inside glioblastoma cells. Cisplatin concentration in the cell nuclei was shown to be 1.5 times greater than in the cytoplasm. In addition, cisplatin traces were not observed in the glioblastoma plasma membrane.^59^ Mohammadi et al. showed that cisplatin treatment during 3 h of PC12 (phaeochromocytoma) cells significantly affected the content of various lipids and their derivatives, in particular, phosphatidylcholine and cholesterol, which were both depleted.^60^ In this study, we found a decrease in the amount of phosphatidylcholine during 1 h incubation with cisplatin, but this difference was not statistically significant. It has been suggested that such lipid changes are involved, at least in part, in the regulation of exocytosis of cisplatin.

A second possible reason for membrane viscosity alterations in treated cells could be a direct interaction of cisplatin with plasma membrane. However, the data about mechanistic interactions of cisplatin with membrane lipids and their consequences for viscosity are quite controversial. In the work by Wang et al.,^61^ it was shown that platinum-containing preparations increase the order of lipid membranes, and lateral lipid diffusion is reduced, which leads to an increase in the viscosity of the membrane. The type and number of anionic lipids were believed to be responsible for this effect. The interaction of cisplatin with phospholipids is specific for negatively charged phospholipids and takes place at low chloride ion concentration.^62^ On the other hand, cisplatin can interact with the hydrocarbon tails of phospholipids, which are part of the membrane.^63^ The binding of cisplatin to the carboxylate and/or phosphate groups of the lipid tails causes an increase in the intermolecular distances of the acyl chains, which can result in decrease of the membrane viscosity. The reported differences can be attributed to different compositions of membranes in different cells lines or different designs of the studies. To test if the effects of direct drug–membrane interaction contribute to the viscosity changes induced by cisplatin, we analyzed the viscosity of lipid vesicles of three different compositions upon interaction with cisplatin (Fig. S4 in the Supplemental Materials). We detected no changes in the lifetime of molecular rotor upon prolonged exposure to cisplatin. Although these lipid preparations did not contain anionic lipids, we tested three compositions with viscosities ranging from 155 to 435 cP, which includes the observed membrane viscosity.

Additionally, we performed cisplatin experiments on two different cell lines and tested different doses of cisplatin. No cell-line-dependent alterations in viscosity were detected. We also found that cisplatin in the range of the concentrations 1 to 4  μM does not display any dose-dependent effects on cell membranes of HeLa Kyoto cells.

Several papers demonstrate that cisplatin induces the formation of reactive oxygen species (ROS) in cells resulting, among other effects, in lipid peroxidation, although this is less appreciable effect in terms of its cytotoxicity compared to DNA damage.^17^^,^^64^ Lipid peroxidation directly affects the double bond of unsaturated fatty acids, leading to changes of the membrane structure.^65^ Our previous experiments using molecular rotors demonstrated that membrane viscosity in model lipid bilayers increases significantly as a results of type II ROS production (singlet oxygen).^66^ However, type I ROS production (radical products) led to viscosity decrease in model membranes containing unsaturated phospholipids.^66^ A large viscosity increase *in cellulo* was previously shown in cancer cells during photodynamic therapy that is known to cause cell death via production of ROS by a photosensitizer.^67^^,^^68^ Therefore, cisplatin-induced lipid peroxidation could also contribute to the increase in membrane viscosity in cisplatin-treated cells and spheroids; this possibility requires further investigation.

Most human cancers quickly become insensitive to the cytotoxic effects of cisplatin due to acquisition of drug resistance. Several studies have suggested that altered membrane properties might be responsible for the acquired resistance to cisplatin treatment. Differences in the viscosity of membranes in tumor cells with different sensitivities to cisplatin have been reported in several papers. For example, Liang et al. used a fluorescent conjugate of cisplatin to demonstrate that the fluidity of membrane lipids decreased in resistant A549/DDP cells compared to sensitive pulmonary adenocarcinoma A549 cells.^13^ Huang et al.^48^ with the use of fluorescence probe TMA-DPH and ^1^H and ^31^P-NMR spectroscopy have found that the microviscosity of the plasma membrane of cisplatin-resistant A549/DDP cells is higher than that of chemosensitive version, which was associated with changes in phospholipid components of the plasma membranes. Cisplatin-resistant breast cancer cells MCF-7/S were also shown to have a different lipid profile compared to sensitive MCF-7/CP cells with higher cholesterol, sphingomyelin, phosphatidylglycerol, and phosphatidylserine levels reported, and reduced levels of phosphatidylcholine and phosphatidylethanolamine.^69^ An increase in cholesterol and sphingomyelin with a decrease in the phosphatidylcholine/sphingomyelin ratio convincingly indicates an increase in the viscosity of the membranes of resistant cells, which is observed for A549/DDP cells above. It was also shown that the microviscosity of the plasma membrane of multidrug-resistant cells is more heterogeneous as compared to nonresistant tumor cells.^70^ It is assumed that increased microviscosity and heterogeneity of the lipid composition of the membranes of tumor cells are important for the manifestation of chemoresistance. Our data detected the increased microviscosity in chemoresistant (cisplatin-adapted) HeLa cells, and this result is consistent with other studies.^48^^,^^69^ Similar alterations were observed in plasma membranes of all cells after the prolonged (>24  h) treatment with cisplatin, which allowed us to assume that this nonspecific cell response can be a mechanism of cells adaptation to the drug. It is not clear though what triggers the adaptive changes in lipid composition and subsequent modification of membrane physical properties. Based on our tests on model lipid membranes in the presence of cisplatin, we can rule out direct mechanistic interactions of cisplatin with cell membrane, however, we do not have any evidence, in which of the more complex cascades of physiological reactions, e.g., DNA damage, oxidative stress, signaling pathways can play a role.

Little is known about the regulation of membrane viscosity in response to cellular stress. Therefore, our findings indicate that membrane lipids in cancer cells have rather dynamic organization. An adjustment of membrane viscosity through changes in lipid composition in response to chemotherapeutic intervention allows cells to maintain cellular homeostasis and survive. These changes in membrane properties can be used as an advantage to develop specific and targeted therapies aimed at plasma membrane of cancer cells and also to increase the effectiveness of the drugs.

## 5 Conclusions

In summary, our results indicate that: (1) microviscosity of the plasma membrane in cancer cells increases after chemotherapy with cisplatin, when used at therapeutically relevant concentrations. (2) This effect is common for two different cancer cell lines (HeLa and CT26) in monolayers and also observed for tumor spheroids. (3) All live treated cells irrespective of their further fate—cell survival or cell death, and in the drug-adapted cell line display increased viscosity. We hypothesize that increased viscosity can be a general mechanism of adaptive response to the drug. (4) The changes in membrane viscosity are associated with changes in lipid composition.

Given that the cell plasma membrane is directly involved in drug transport and regulation of numerous biological processes, additional knowledge about the effects of therapeutic agents on its physical properties may help to design more effective treatment regimens and better understand the mechanisms of drug resistance.
