# Supplementary material for: Mapping cisplatin-induced viscosity alterations in cancer cells using molecular rotor and fluorescence lifetime imaging microscopy
Source: J Biomed Opt. 2020 Dec 16;25(12):126004. doi: 10.1117/1.JBO.25.12.126004 (PMC7744042; doi:10.1117/1.JBO.25.12.126004)
Supplement: Supplementary file 1 [file JBO_025_126004_SD001.pdf]

Supplementary information

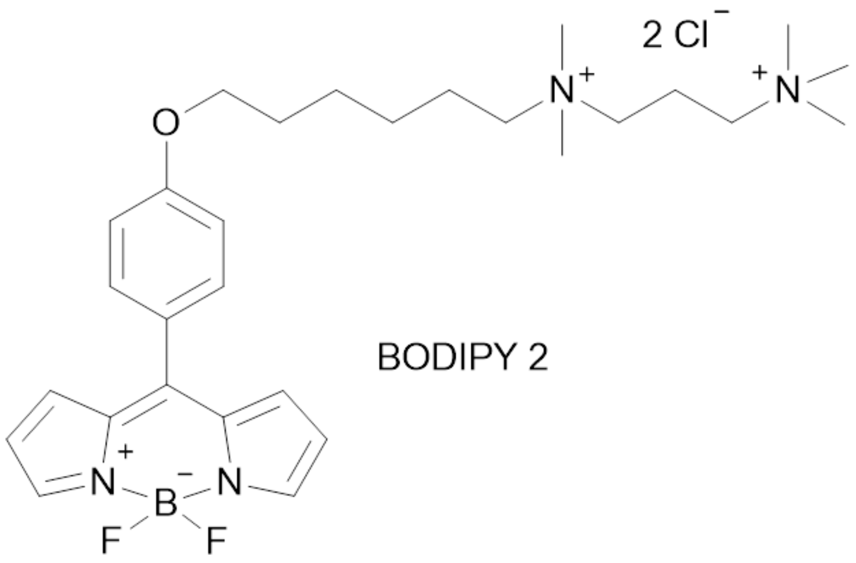

Fig. S1 The molecular structures of molecular rotors BODIPY 2.

As shown in figure S2, the molecular rotor is internalized into dead cells (PI positive), where its fluorescence decayed bi-exponentially, indicative of rotor aggregation. This could be seen from high χ2 value of a monoexponential fitting (blue color). The presence of aggregates causes the biexponential decay kinetics and it becomes impossible to interpret the changes in fluorescence lifetime of the rotor as viscosity changes[43].

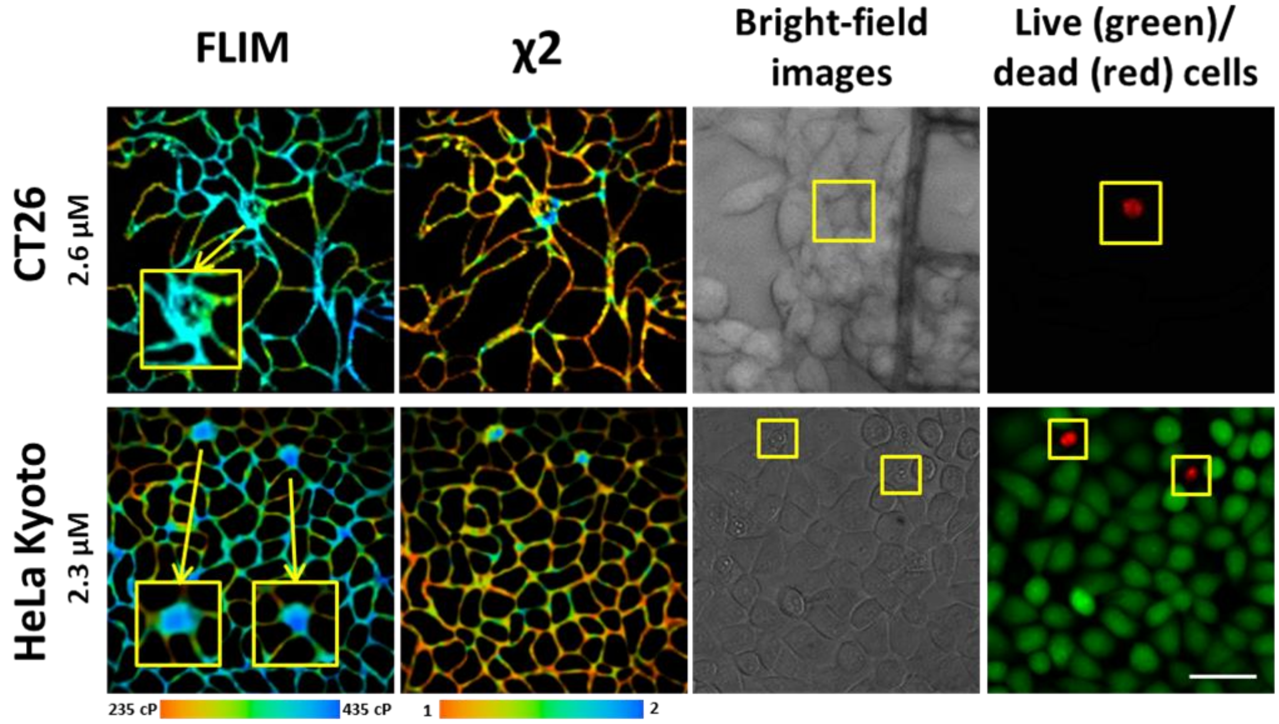

Fig. S2 Representative images of microviscosity in monolayer CT26 (top row) and HeLa Kyoto (bottom row) cells during 24 hour-exposure to cisplatin. Viscosity images of cells aquired with FLIM of fluorescent molecular rotor BODIPY 2 (monoexponential decay model, fluorescence lifetime (left column) and χ2 (second column) are shown), bright-field images (third column), visualization of live and dead cells stained with calcein (green) and propidium iodide (red) (fourth column). Scale bar, applicable to all images, is 40 µM.

Representative fluorescence decay curves of fluorescent molecular rotor BODIPY 2 in membrane of cancer cells are shown in Fig.S3.

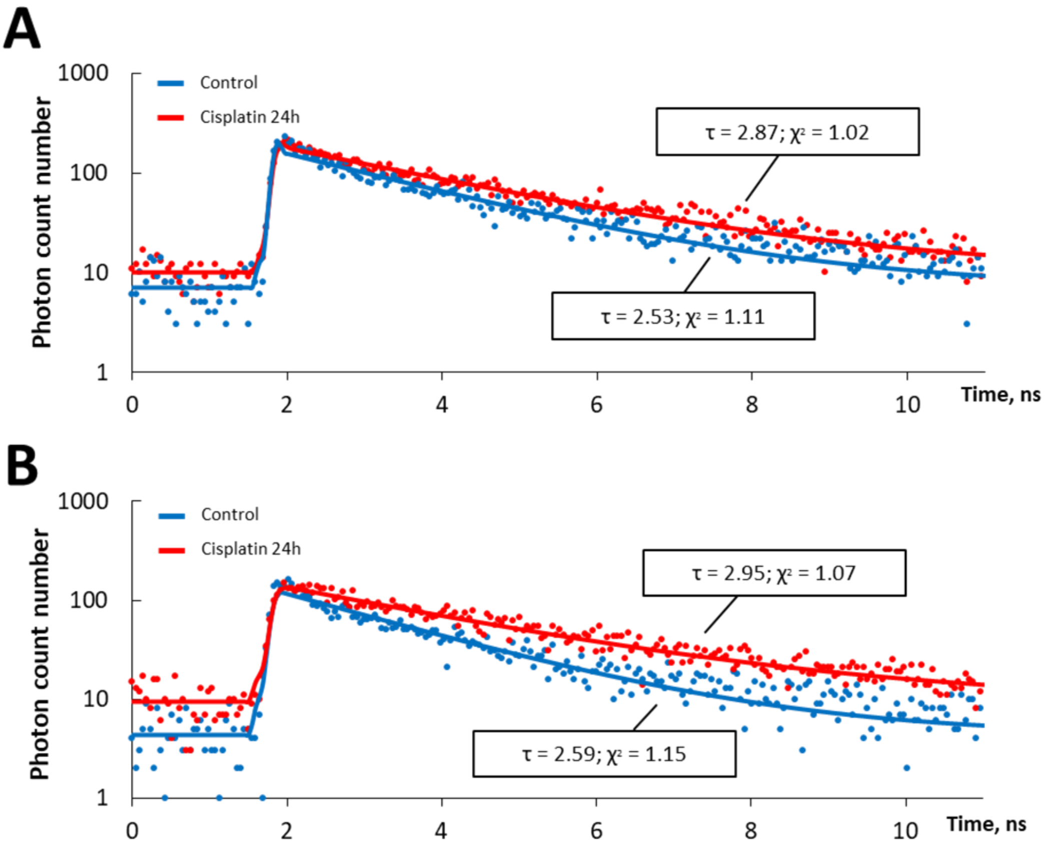

Fig. S3 Fluorescence decay curves of fluorescent molecular rotor BODIPY 2 in the specific spot in the membrane of control and cisplatin-treated cancer cells. Mono-exponential fits for control (blue curve) and cisplatin-treated (red curve) CT26 cells (A) and HeLa cells (B) in cell monolayers.

Figure S4 demonstrates that cisplatin does not alter the viscosity in an aqueous suspension of LUVs.

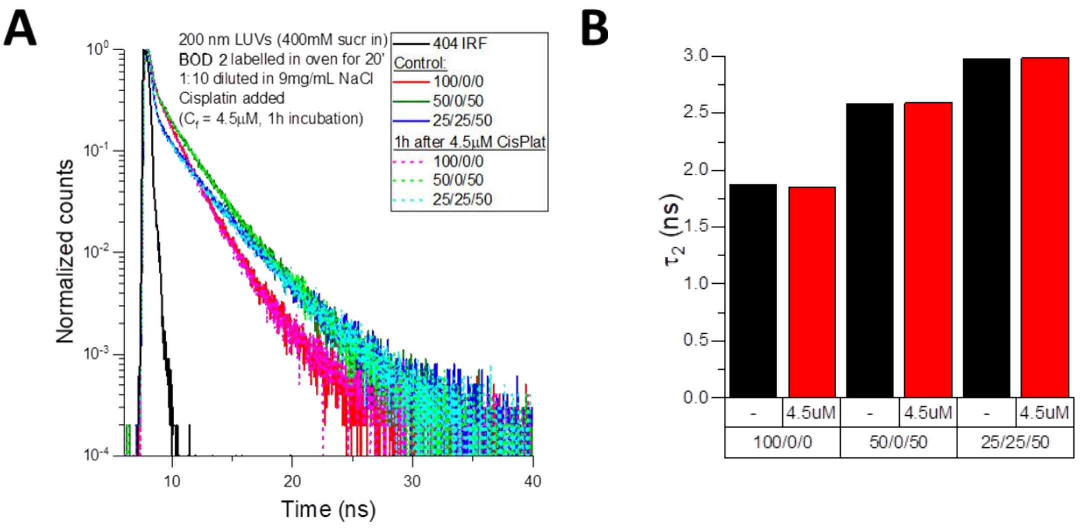

Fig. S4 Microviscosity of LUVs produced from DOPC, DOPC + Cholesterol (50/50) and DOPC/ Sphingomyelin/Cholesterol (25/25/50) after 1 h incubation with cisplatin. (A) Time resolved fluorescence decay traces recorded for BODIPY 2 in corresponding LUVs before (solid lines) and after (dashed lines) addition of cisplatin. The short lifetime component corresponds to non-internalized BODIPY 2, while the

longer component corresponds to the embedded rotor [Dent et al. PCCP 2015]. (B) The fitting results indicate that no significant changes in lifetime were observed 1 h after cisplatin addition.

After removing cisplatin from the culture medium, we measured the viscosity after another 48 hours. Figure S5 shows that the cells retained the increased viscosity of their plasma membranes.

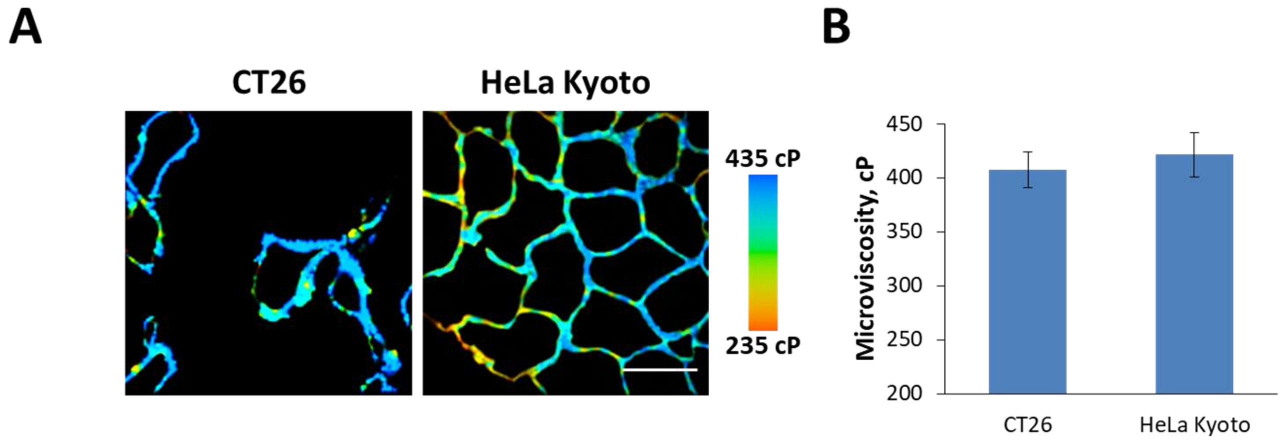

Fig. S5 Membrane microviscosity of CT26 and HeLa Kyoto cells 48 h after removal of cisplatin from the culture medium. (A) Viscosity images of cells aquired with FLIM. (B) Quantification of viscosity of CT26 and HeLa cells after removal of cisplatin from the culture medium. Mean ± SD, n = 20 cells. Bar, 30 μM.

We investigated therapeutic effects of cisplatin on tumor spheroids. It was found that cisplatin treatment did not affect the size of the spheroids while increasing the number of dead cells (Fig. S6).

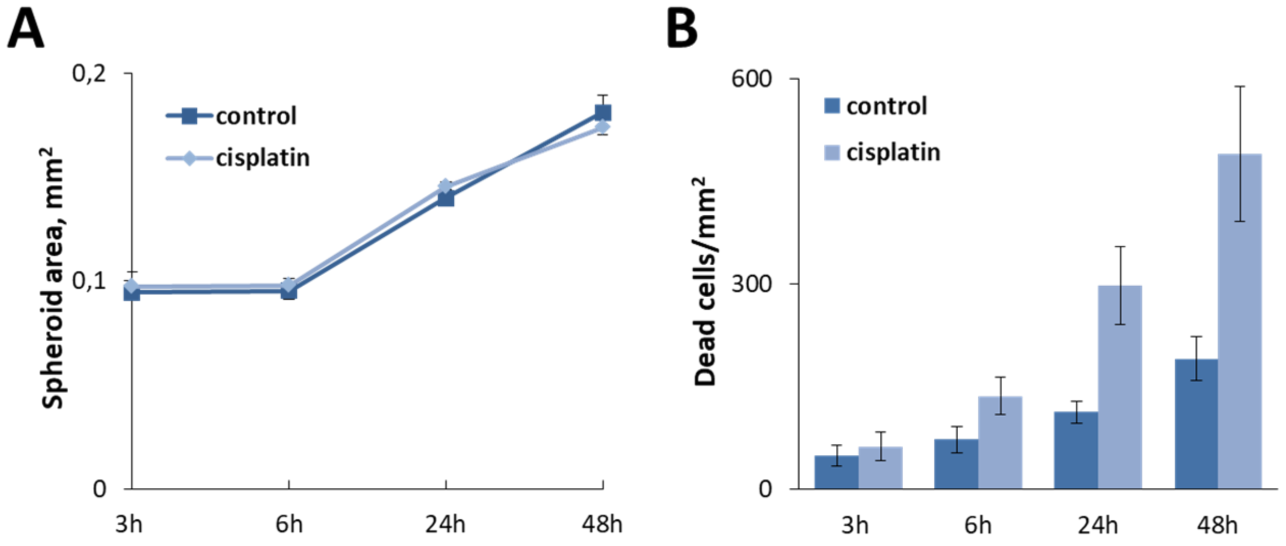

Fig. S6 Effects of cisplatin on tumor spheroids. (A) Growth curves for untreated control and cisplatin-treated spheroids. (B) Quantification of dead (PI-stained) cells in the tumor spheroids.

Representative fluorescence decay curves of fluorescent molecular rotor BODIPY 2 in membrane of cisplatin-adapted HeLa Kyoto cells are shown in Fig.S7.

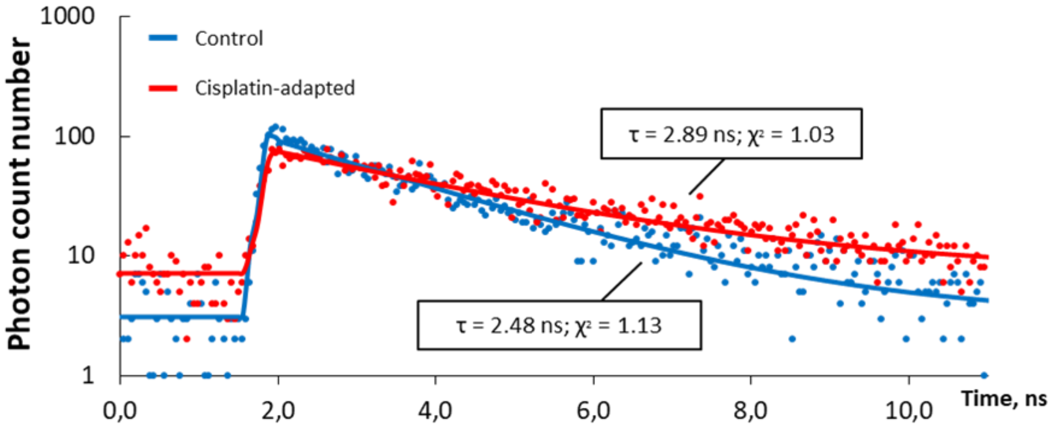

Fig. S7 Fluorescence decay curves of fluorescent molecular rotor BODIPY 2 in the specific spot in the membrane of control and cisplatin-adapted cancer cells. Mono-exponential fits for control (blue curve) and cisplatin-adapted (red curve) HeLa cells.
